# Evaluation of a geriatrics primary care model using prospective matching to guide enrollment

**DOI:** 10.1186/s12874-021-01360-4

**Published:** 2021-08-16

**Authors:** Valerie A. Smith, Courtney Harold Van Houtven, Jennifer H. Lindquist, Susan N. Hastings

**Affiliations:** 1 Center of Innovation to Accelerate Discovery and Practice Transformation (ADAPT), Durham Veterans Affairs Health Care System, 411 W Chapel Hill St Suite 600, NC 27701 Durham, USA; 2 Department of Population Health Sciences, Duke University School of Medicine, 411 W Chapel Hill St Suite 600, NC Durham, USA; 3 Department of General Internal Medicine, Duke University, 411 W Chapel Hill St Suite 600, NC Durham, USA; 4 Duke-Margolis Center for Health Policy, Durham, USA; 5 Center for the Study of Aging and Human Development, Duke University School of Medicine, NC Durham, USA; 6 Department of Medicine, Division of Geriatrics, Duke University School of Medicine, NC Durham, USA; 7 Geriatrics Research Education and Clinical Center (GRECC), Durham Veterans Affairs Health Care System, NC Durham, USA

**Keywords:** Prospective study design, Exact matching, Distance function matching, Comparative effectiveness, Average treatment effect, Geriatrics

## Abstract

**Background:**

Few definitive guidelines exist for rigorous large-scale prospective evaluation of nonrandomized programs and policies that require longitudinal primary data collection. In Veterans Affairs (VA) we identified a need to understand the impact of a geriatrics primary care model (referred to as GeriPACT); however, randomization of patients to GeriPACT vs. a traditional PACT was not feasible because GeriPACT has been rolled out nationally, and the decision to transition from PACT to GeriPACT is made jointly by a patient and provider. We describe our study design used to evaluate the comparative effectiveness of GeriPACT compared to a traditional primary care model (referred to as PACT) on patient experience and quality of care metrics.

**Methods:**

We used prospective matching to guide enrollment of GeriPACT-PACT patient dyads across 57 VA Medical Centers. First, we identified matches based an array of administratively derived characteristics using a combination of coarsened exact and distance function matching on 11 identified key variables that may function as confounders. Once a GeriPACT patient was enrolled, matched PACT patients were then contacted for recruitment using pre-assigned priority categories based on the distance function; if eligible and consented, patients were enrolled and followed with telephone surveys for 18 months.

**Results:**

We successfully enrolled 275 matched dyads in near real-time, with a median time of 7 days between enrolling a GeriPACT patient and a closely matched PACT patient. Standardized mean differences of < 0.2 among nearly all baseline variables indicates excellent baseline covariate balance. Exceptional balance on survey-collected baseline covariates not available at the time of matching suggests our procedure successfully controlled many known, but administratively unobserved, drivers of entrance to GeriPACT.

**Conclusions:**

We present an important process to prospectively evaluate the effects of different treatments when randomization is infeasible and provide guidance to researchers who may be interested in implementing a similar approach. Rich matching variables from the pre-treatment period that reflect treatment assignment mechanisms create a high quality comparison group from which to recruit. This design harnesses the power of national administrative data coupled with collection of patient reported outcomes, enabling rigorous evaluation of non-randomized programs or policies.

## Background

In the Department of Veterans Affairs (VA) health care system, primary care is provided through Patient Aligned Care Teams (PACTs) based on a patient-centered medical home model of team-based primary care.[1] GeriPACT is a “Special Population PACT” designed to provide comprehensive primary care combined with specialty expertise for complex geriatric and high-risk Veterans.[2, 3] GeriPACT integrates and coordinates traditional ambulatory health care services with a variety of community-based services. Specifically, the structure of GeriPACT differs from traditional PACT in three key ways: 1) providers have geriatrics expertise;  2) emphasis on interdisciplinary team care, e.g. each GeriPACT team includes a pharmacist and social worker; and3) smaller panel sizes (2/3 of usual PACT size)[3].

In this way, GeriPACT strives to optimize independence, quality of life, and quality of care for Veterans who are particularly vulnerable due to multiple interacting cognitive, functional, psychosocial, and medical challenges in the setting of advanced age. However, we lack essential information on the patients’ perspectives of how quality and experience of care differ after a patient moves from usual care, in PACT, to GeriPACT. Understanding the relative value of existing primary care delivery models is of great relevance as accountable care organizations and health systems look for effective ways to improve quality and value for older adults.

To understand the effect of GeriPACT on patient experience and key quality of care clinical process measures compared to traditional PACT, we are conducting a prospective study comparing outcomes over an 18 month observation period. Randomized assignment of patients to GeriPACT vs. a traditional PACT was not feasible because GeriPACT was established by VA directive as a new model of care. As such, there was no way to influence the rollout in a randomized design, and the decision to transition to GeriPACT is made jointly by a patient and provider. For example, there may be a pattern of escalating hospitalizations or indications of dementia that lead a primary care physician to refer a patient to GeriPACT. Additionally, full evaluation of the impact of this care model requires understanding the patient’s perspective of their care beyond constructs measured in the electronic health record. Thus, to accurately evaluate the GeriPACT healthcare model, the study design needed to incorporate two crucial features: a non-randomized comparable group of patients not receiving care from GeriPACT for a control group and the ability to collect patient reported outcomes not available via administrative data.

In this paper, we describe and present the conceptual motivation for our study design. We demonstrate the feasibility of our approach for prospectively matching and enrolling participants, and provide information on our success at minimizing confounding (threats to internal validity) using the characteristics of the fully recruited baseline cohort. Finally we discuss advantages and limitations of our study design and provide practical guidance for researchers interested in implementing a similar approach in other studies.

## Methods

### Overview of study design

Our overall study objective was to examine the average treatment effect of the GeriPACT model across VA on an array of patient-reported and administratively measured outcomes. To meet this objective, we prospectively matched and enrolled GeriPACT patients nationwide to PACT patients who used the same facility and were otherwise similar, but whose care remained in traditional PACT. Sample size calculations based on simulations suggested we needed 275 dyads enrolled to have adequate power to detect differences across outcomes (details below). We consulted a panel of experts in geriatric care, including study team members, clinicians, and partners in VA’s Office of Geriatrics and Extended Care, to construct a comprehensive list of confounding patient and facility characteristics that could influence the relationship between transferring care to GeriPACT and patient outcomes. The extensive information we had on the treatment assignment mechanism was critical to the validity of the proposed approach.

We focused on patients with at least 2 visits to GeriPACT and matched on identified important pre-exposure health and utilization characteristics. Matching on pre-exposure characteristics reflects the patient’s health and use during the period of *referral* to GeriPACT. In this way, we can compare similar patients who either (1) transferred care to GeriPACT; or (2) continued to receive usual care in PACT. Following enrollment, longitudinal patient reported outcomes were collected via telephone surveys at baseline and continue to be collected at 3 subsequent time points (6, 12, 18 months) to obtain patients’ quality of life and experience of care. We required all patients be community-dwelling, not institutionalized or receiving Hospice or palliative care.

### Outcomes

We collected a combination of patient-reported via survey and electronic health record-derived patient outcomes to compare the experiences of patients in GeriPACT compared to those in PACT. Specifically, we focused on two domains: quality of care and patient experience outcomes. Our primary outcome, a patient experience outcome, was patient days at home, defined as days alive and neither in an emergency department (ED) nor inpatient setting. Secondary outcomes include potentially inappropriate medications, presence of advance directive, frail elderly performance measures, patient’s care integration, and self-reported health and wellbeing.

### Sample size calculations

The power calculation was based on the primary hypothesis that patients who transfer care to GeriPACT will experience a greater number of days at home than those in PACT. Power estimates were generated empirically via simulation in SAS. We generated 1,000 simulated datasets assuming the data followed both a zero-inflated Poisson (ZIP) or zero-inflated negative binomial distribution (ZINB). We anticipated PACT patients to average 5.5 days not at home and GeriPACT patients to average 4 days not at home over the 18-month outcomes interval, reflecting a 1.5 day mean reduction. From running the simulations and accounting for clustering among patients seen at the same VAMC, we determined that a sample size of 550 patients (275 dyads) would provide 90 % power should the data follow a ZIP distribution and at least 80 % power should it follow a ZINB distribution with variance up to 12 times larger.

### Setting and population

#### Defining the sample

Subjects were selected from VA medical centers nationally with ≥ 500 GeriPACT visits in fiscal year 2016. Following guidance from VA operations, we defined GeriPACTs based on three criteria: (1) use of clinic stop code 350; (2) maximum panel size of 800 unique patients per 1 provider full time equivalent; and (3) include a social worker and pharmacist as team members.

**Fig. 1:**
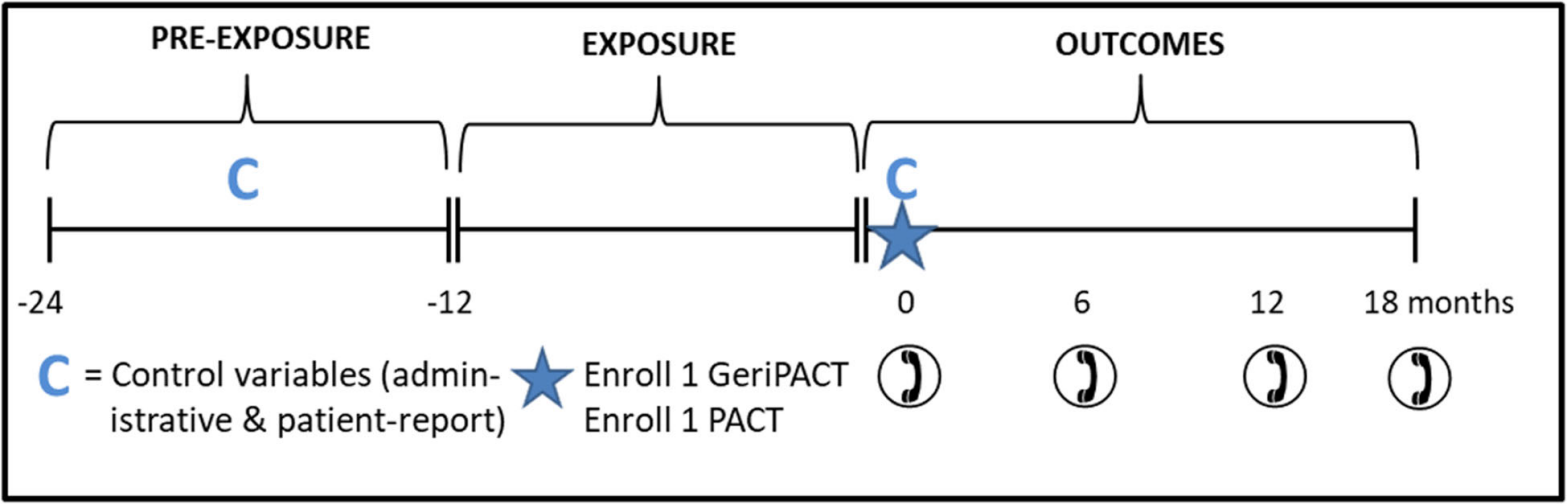
Study Flow Timeline

A rolling data pull, updated quarterly, identified potentially eligible patients once they had ≥ 2 PACT visits and no GeriPACT visits in the 1-year pre-exposure period and ≥ 2 GeriPACT visits and no PACT visits in the 1-year exposure period (Fig. 1). This enabled us to construct a cohort of patients who transferred from PACT to GeriPACT. On the same schedule, patients were identified as a potentially eligible PACT patient once they had ≥ 2 PACT visits and no GeriPACT visits in both the pre-exposure and exposure period.

#### Matching procedures

From a quarterly pull of potentially eligible patients, we performed matching to construct pools consisting of 1 GeriPACT patient and multiple similar PACT patients from which to recruit. The matching characteristics were derived from the 12 months prior to the exposure period (pre-exposure period in Fig. 1); thus, these characteristics were fixed before GeriPACT enrollment. We then used the identified confounders in a combination of coarsened exact and minimum Mahalanobis distance function matching. The mix of matching approaches allowed us to utilize a nonparametric method of preprocessing data to control for the potentially confounding influence of pretreatment control variables. Specifically, we created pools of eligible matches (PACT patients) for each GeriPACT patient matching on the following “coarsened” characteristics: primary site at which care was received, age within 5 years, race, sex, presence of advance directive, dementia diagnosis in the pre-exposure period, and whether the patient had a hospitalization in the prior year. We further refined the matched by selecting those who were closest to the GeriPACT patient on the basis of a Mahalanobis distance function, which calculated the closeness of continuous variables after standardizing them.[4] We matched one GeriPACT patient to at least five PACT patients whose distance function was closest to the GeriPACT patient based on age, count of hospitalizations, Care Assessment Need (CAN) score, and JEN Frailty Index (JFI) score, to reflect aggregate measures of clinical conditions and medical needs in efforts to minimize confounding. CAN scores predict hospitalization and mortality, and range from 0 to 99 where patients with larger values are more likely to experience hospitalization or death within a year.[5] JFI predicts institutional care and other home-based care and services, and ranges from 0 to 13, with higher scores reflecting higher risk of use.

We required there to be *at least* 5 PACT matches per GeriPACT patient due to an expected recruitment rate of 20 %, based on our prior studies utilizing telephone-based recruitment, and excluded GeriPACT patients without at least 5 matches. In other words, we anticipated the potential need to contact 5 PACT patients in order to enroll one. We deemed the potential exclusion of the portion of the GeriPACT population without 5 matches acceptable because the slight loss of generalizability was outweighed by the internal validity accomplished by matching on the selected characteristics.

#### Recruitment and enrollment procedures

**Fig. 2:**
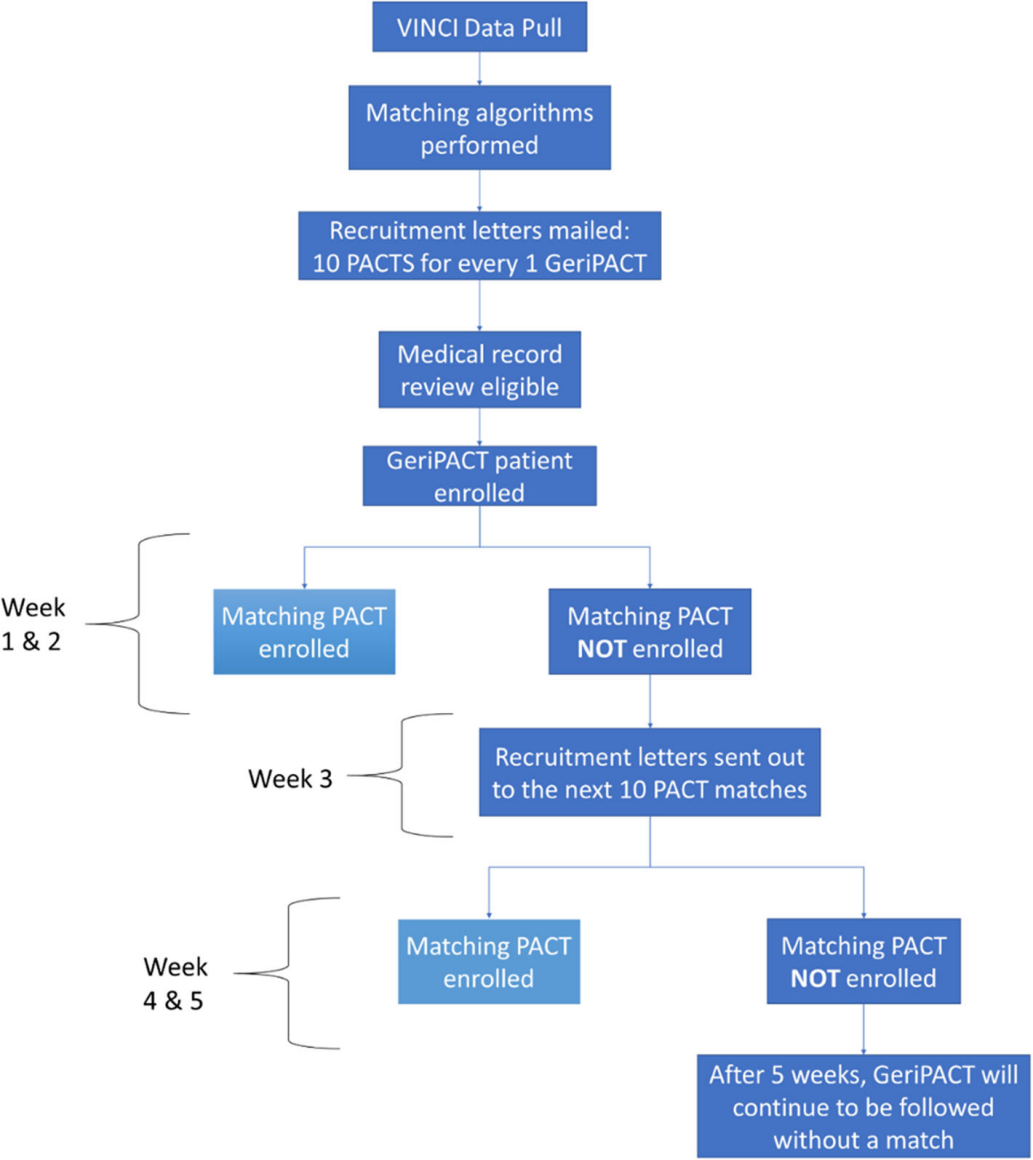
Flowchart of Patient Recruitment Procedures

Patients were recruited from VA facilities nationally with fully implemented GeriPACTs, using VA administrative data to form pools of eligible GeriPACT and PACT matched pairs. Figure 2 displays the matching and patient recruitment process. After matching, letters were sent to GeriPACT patients and their closest matches (up to 20 if available), to notify them of our study. After medical record review to confirm eligibility, we then contacted potentially participants by telephone to obtain verbal consent and enroll them in the study.

We first enrolled a GeriPACT patient and then proceeded with targeted recruitment for that patient’s match. After a GeriPACT patient was enrolled, we utilized the pool of all matches available created by coarsened exact matching for that patient to prioritize our recruitment attempts. Recalling that we prioritized recruitment of PACT patients based on the distance function, we grouped standardized differences into ranges we considered essentially equivalent for the purpose of comparability to prioritize recruitment. For example, the highest priority matches to enroll were those PACT patients with distance functions < 0.1 from the GeriPACT patient, the second highest were those with distance functions < 0.25, the third with distance functions < 0.5, and so on. When a given GeriPACT patient had many equivalent potential matches, this allowed efficient recruitment by attempting to contact multiple potential matches when eligible patients were unable to be contacted immediately (e.g., when there was no answer by phone).

We attempted to enroll potential matches in the closest “priority” group before moving on to attempt enrollment of matches with larger distance function differences. We started with the closest priority groups, and after two weeks if no match had been enrolled, expanded recruitment to lower prioritized match groups. Importantly, we considered all PACT patients who met the match criteria to be suitable matches regardless of the priority group, so we continued to recruit through the entire pool if needed.

#### Data collection procedures

After enrollment, baseline patient information was collected via telephone survey. Because matching occurred prior to telephone contact, these self-reported measures were unavailable to utilize in the matching process. However, they could be important to use for adjustment as appropriate as control variables; for example, presence of an informal caregiver and number of individuals in the household. Thus, in addition to matching on important pre-exposure characteristics, outcome regression models can then include control variables from two key sources: (1) administrative and medical records in the pre-exposure period and (2) the baseline survey at the time of enrollment for factors not influenced by exposure to treatment. Patient-reported outcomes were collected via telephone surveys at 6, 12 and 18 months following enrollment (data collection ongoing and thus not reported in this manuscript).

## Results

We identified 4,925 unique GeriPACT patients eligible for enrollment from 57 eligible VA facilities. Of those patients, 647 (13.1 %) lacked 5 suitable matches and thus we did not attempt to enroll them. Among those with at least 5 matches, the median number of available matches from which to recruit was 129 per GeriPACT patient, with a range of 5 to 3,848.

**Fig. 3:**
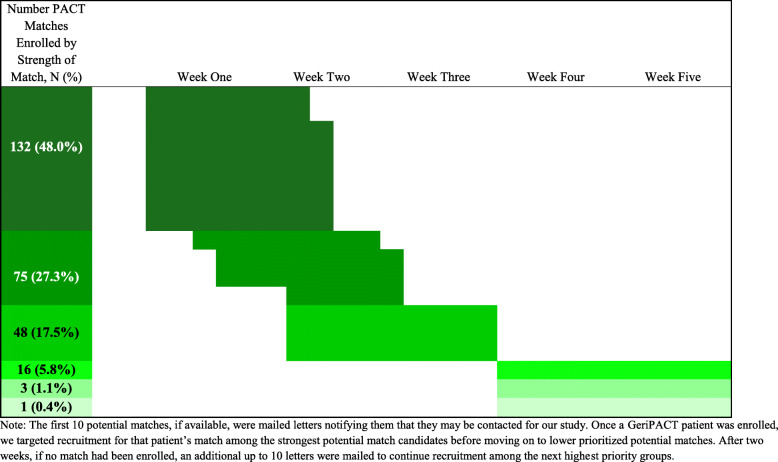
Proportion of Sample Recruited by Strength of Match based on Prioritization Schedule for Recruitment Efforts of Potential Matches by Week of Recruitment, starting with enrollment of the GeriPACT patient

Recruitment was completed in December 2019 when we reached our targeted sample size of 275 matched GeriPACT-PACT dyads. Because we recruited GeriPACT patients before recruiting their matches, we enrolled a total of 292 GeriPACT patients and enrolled a suitable match for 275 of them (94 % of enrolled GeriPACT patients). We successfully enrolled 48 % of matches in the closest possible priority group and 27 % in the second closest (Fig. 3). More than 98 % of enrolled matches have come from the four closest possible priority groups available for each GeriPACT patient, and the median days between enrollment of the GeriPACT patient and their representative match is 7.

### Baseline characteristics

Table 1 presents baseline characteristics of the enrolled GeriPACT patients and their PACT matches, along with standardized mean differences (SMDs). SMDs < 0.2 in absolute value indicate reasonable covariate balance and are not sensitive to sample size.[6] On average, patients in both arms are older (mean age 81 and 79 for GeriPACT and PACT patients, respectively), predominantly male (98.2 %) and white (83.3 %). Only a small fraction (3.3 %) had dementia diagnoses at baseline and mean CAN scores were 49.4 and 53.1, for GeriPACT and PACT patients, respectively, close to 50, the median nationwide percentile. Mean JFI scores were also similar at around 4, indicating that patients in both groups were of moderate frailty. Scores of ≥ 7 are considered “high frailty.”

**Table. 1:** Baseline Characteristics of Matched GeriPACT and PACT Patients in Veterans Affairs Facilities in 2016

Patient Characteristic	GeriPACT (*n* = 275)	PACT (*n* = 275)	Standardized Mean Difference
*Data Collected from Electronic Health Record, used in matching*			
Age, mean (SD)	80.8 (6.9)	79.4 (7.0)	-0.20
Black race, N (%)	34 (12.4 %)	34 (12.4 %)	0
White race, N (%)	229 (83.3 %)	229 (83.3 %)	0
Other non-white race or missing, N (%)	12 (4.4 %)	12 (4.4 %)	0
Female, N (%)	5 (1.8 %)	5 (1.8 %)	0
Presence of note related to advance directive, N (%)	77 (28.0 %)	77 (28.0 %)	0
Any Hospitalizations in prior year, N (%)	22 (8.0 %)	22 (8.0 %)	0
# of Hospitalizations in prior year, mean (SD)	0.11 (0.43)	0.10 (0.40)	-0.03
JEN Frailty Index (JFI) score, mean (SD)	4.0 (2.0)	3.9 (1.8)	-0.05
Care Assessment Need (CAN) score, mean (SD)	49.4 (32.1)	53.1 (29.4)	0.12
Dementia diagnosis, N (%)^1^	9 (3.3 %)	9 (3.3 %)	0
*Measures Collected via Baseline Survey, not used in matching*			
Cognitive status (education-adjusted TICS-m) mean (SD)^2^	30.2 (4.6)	30.4 (5.1)	0.06
Number of ADLs with help needed, mean (SD)	1.4 (1.7)	1.0 (1.4)	-0.23
Number of IADLs with help needed, mean (SD)	2.2 (2.2)	1.7 (2.0)	-0.23
Social support mean (SD)^3^	49.4 (10.0)	50.4 (10.4)	0.09
Good or Okay Financial security N(%)^4^	189 (68.7 %)	215 (78.2 %)	0.19
Inadequate health literacy N (%)^5^	73 (26.5 %)	74 (26.9 %)	-0.02
Number of individuals in household, mean (SD)	1.9 (0.9)	1.9 (0.8)	0.04
Presence of informal caregiver, N (%)^6^	153 (55.6 %)	160 (58.2 %)	0.05

^1^ Not measured in first data pull wave, and thus not collected for *n* = 56 patients in each arm

^2^* n* = 24 missing values in GeriPACT arm; *n* = 13 missing in PACT arm

^3^* n* = 6 missing values in GeriPACT arm; *n* = 8 missing in PACT arm

^4^* n* = 6 missing values in GeriPACT arm; *n* = 1 missing in PACT arm

^5^* n* = 25 missing values in GeriPACT arm; *n* = 13 missing in PACT arm

^6^* n* = 1 missing value in GeriPACT arm

Among survey-collected baseline measures, characteristics are quite similar by treatment group. For example, cognitive status scores of 30.2 and 30.4 were remarkably similar. Interpretation of this score depends on many factors such as education and age, but in our study, we classify those with a modified version of the Telephone Interview for Cognitive Status (TICS-m) education-adjusted score of ≤ 27 as having significant cognitive impairment.[7] The sample overall had evidence of functional disability, whereby both groups reported needing help with 1 activity of daily living (ADL) on average and help with 2.2 to 1.7 instrumental activities of daily living (IADLs) on average, by GeriPACT or PACT status. The mean social support score was 49–50 for both groups. A higher score reflects more social support on domains such as emotional support, affection, positive interaction, and informational support, and the average social support score reflected here is 20 points lower than in other national populations of chronically ill patients, which have a mean score of 70.[8].

As expected, all variables on which we exact matched had SMDs of zero, and those included in the distance function had small SMDs, exclusively < 0.2 in absolute value (Table 1). In addition to the administrative covariates used in matching, we also collected several important baseline characteristics after matching via patient survey at the time of enrollment. With the exception of functional disability (ADLs and IADLs; SMD = 0.23), which is slightly over the 0.2 threshold, all other survey-collected baseline variables had a SMD ≤ 0.2 in absolute value.

## Discussion

We present an important process to prospectively evaluate treatment effects when randomization is infeasible, unethical, or undesired. This design harnesses the power of nationally collected electronic health record data coupled with collection of patient reported outcomes. The success demonstrated, as evidenced by matched patient recruitment and balance in baseline characteristics, in the implementation of this design to evaluate the GeriPACT model of care suggests that this is a promising approach. The results of our study will address a well-recognized barrier in improving care for older adults- limited guidance about which care models are most effective. For example, if the study demonstrates important differences in quality of care or patient experiences in GeriPACT patients compared to traditional primary care, clinical and health system leaders may choose to allocate resources to expand this model to care for a large and growing population of vulnerable older adults. Additionally, with study-specific modifications, this approach could be applied to future studies seeking to evaluate programs or policies without the benefit of randomization, but also without the loss of the ever-important patient perspective. While our example relates to evaluating a model of care, this approach could be used in a variety of contexts, such as evaluation of caregiver support programs or assessing patient reported outcomes following infection with COVID-19 or other conditions.

Our study design approach of matching prior to enrolling a targeted national sample to evaluate a model of care led to successful baseline covariate balance and patient enrollment. This experience suggests such a study design may be promising in other contexts for evaluation of non-randomized treatments or policies. However, many study-specific design decisions must be made, and these decisions can be further complicated by the uncertainty of whether identified eligible patients will choose to enroll. For example, recruitment required enrolling a GeriPACT patient prior to knowing whether we could successfully enroll one of their suitable matches. While we were able to successfully enroll matches for 94 % of GeriPACT patients, had this rate been lower, internal validity may have faced greater threat. We highlight below some of our significant design decisions and rationale.

With any implementation of coarsened exact matching, there is a risk of losing patients in the treated population due to lack of finding someone who exactly matches the treated patient on all characteristics. With more characteristics on which to exact match, the potential for confounding is minimized and internal validity strengthened. Conversely, the higher the likelihood that no individual exists in the control group with the array of desired characteristics for a match, the lower the generalizability to the entire population of interest. The tradeoff between internal validity and generalizability will need to be weighed for any given study. Determining which characteristics to include can be based on the clinical importance of each characteristic to the outcome process and a consideration of how representative the control group is compared to the treatment group prior to any matching. We suggest testing matching procedures using preparatory data prior to enrollment to evaluate what proportion of treated patients may be lost due to lacking matches with varying combinations of desired covariates. Doing this prior to enrollment allows the study team to optimize the internal vs. external validity tradeoffs prior to outcomes collection. For our study, in which GeriPACT serves a much smaller number of patients than in the usual care PACT group, we were able to include all desired covariates in our matching algorithm while still meeting our recruitment goals for GeriPACT patients. If this were not the case, the study team would have to evaluate which matching criteria can be loosened while still maintaining appropriate control of baseline confounders. Through loosening exact matching criteria, larger pools of potential matches for each treated patient can be created to ensure successful recruitment from both groups.

Relatedly, based on the expected patient recruitment rate, a similar tradeoff informs how many potential available matches are required in order to deem the treated patient eligible for recruitment. We expected 20% of available PACT patients to successfully enroll, and thus we required 5 matches to exist for a treated patient to be eligible, with expectations that this would, for the most part, allow a matched PACT patient to be enrolled for each GeriPACT patient. Lowering this requirement would have excluded fewer treated patients from our sample but could have left a larger proportion without a representative match and could have required additional resources to enroll a greater number of GeriPACT patients to meet the 275 dyad target.

We also decided in advance how long following enrollment of the GeriPACT patient we would attempt to recruit a representative PACT match. Given that the median number of available matches was 129, attempted recruitment of a match often could have continued for months. Due to possible seasonal or time-varying practice effects, we wanted enrollment of the GeriPACT patient and their match to occur as close as possible in calendar time. We determined that 5 weeks was an acceptable window in which to attempt recruitment of a matched PACT patient. Afterwards, if no match was enrolled, the GeriPACT patient remained in the study with no matching PACT patient. The median days between enrollment of the GeriPACT patient and their representative match was 7.0, suggesting our recruitment strategy adequately allowed for matching nearby in calendar time while still prioritizing PACT patients with the closest match. In other studies, the length of this window should be determined by importance of temporal trends and expected ability to recruit matched patients.

Importantly, in addition to the design decisions made, adjustment can be made for unbalanced factors in the analysis phase. For example, data from GeriPACT patients who remained unmatched can be omitted in a sensitivity analysis and additional baseline covariates not included as matching variables can be adjusted for in regressions. Coupling analytic strategies with careful design can strengthen the robustness of conclusions drawn from such a study. Taken together, these procedures optimally balanced our study goals as we felt tight control over our included baseline confounders was crucially important. For different research questions and different populations, it is critical to weigh these decisions as part of designing the study.

Despite the benefits, the prospective approach we designed and used is still subject to several limitations. As in any non-randomized study, it is untestable whether unobserved confounders may exist, so residual confounding may bias treatment effects due to characteristics neither matched on nor collected for adjustment. For example, we did not have access to data on the content of conversations between patients and their providers or patient preference for model of care. Approaches to address unobserved confounding, such as instrumental variables, were not a viable option for identifying individuals to prospectively contact. While matching has been criticized by some[9, 10], matching for prospective enrollment has strong advantages. In our case, balance on post-matching survey-collected measures suggests that matching on the administrative characteristics chosen adequately balanced not only observed, but possibly many unobserved, baseline characteristics. Similarly, despite the prospective design, only data in the electronic health record can be used for matching because survey measures are not collected until enrollment, and it would be impractical to collect survey reported measures on all potential matches in order to refine the match. Additionally, because survey-collected data by necessity occurs after identification of treatment group, and thus after initiation of treatment, measures influenced by receipt of treatment may lie in the causal pathway between treatment and the outcomes of interest. Therefore, adjustment using survey-collected data must be restricted to measures not influenced by treatment. We benefitted from the VA’s extensive nationwide electronic health record data, allowing us to control for documented comorbidities and other variables that may also influence outcomes (e.g. CAN score, JFI, prior hospitalizations). Studies in other settings may be limited by less data availability if conducted in a smaller or more restrictive data environment. Additionally, the population reflected in our data source is predominantly white and male. In future studies, the described prospective matching approach could be coupled with oversampling less represented populations to ensure diverse perspectives are captured.

## Conclusions

Limitations notwithstanding, we believe the presented prospective approach provides the opportunity to increase robustness of policy and program evaluation through strong internal validity and by incorporating crucial patient-reported measures as outcomes. Understanding of the patient perspective is often lacking in rigorous nationwide evaluations. We encourage future studies to consider this approach to achieve a richer understanding of the programs and policies under evaluation.

## Data Availability

The datasets analyzed are available from the corresponding author on reasonable request.

## Abbreviations

GeriPACT: Geriatrics Patient Aligned Care Team
PACT: Traditional Patient Aligned Care Team
VA: Veterans Affairs
CAN Score: Care Assessment Needs Score
JFI: Jen Frailty Index
SMD: Standardized Mean Difference
ADL: Activities of Daily Living
IADL: Instrumental Activities of Daily Living
ZIP: Zero-inflated Poisson
ZINB: Zero-inflated negative binomial
